# A High Precision and Multifunctional Electro-Optical Conversion Efficiency Measurement System for Metamaterial-Based Thermal Emitters

**DOI:** 10.3390/s22041313

**Published:** 2022-02-09

**Authors:** Heng Liu, Meng Zhao, Yongkang Gong, Kang Li, Cong Wang, Yuchen Wei, Jun Wang, Guozhen Liu, Jinlei Yao, Ying Li, Zheyi Li, Zhiqiang Gao, Ju Gao

**Affiliations:** 1 Jiangsu Key Laboratory of Micro and Nano Heat Fluid Flow Technology and Energy Application, Suzhou University of Science and Technology, Suzhou 215009, China; 1911086004@post.usts.edu.cn (H.L.); liying@post.usts.edu.cn (Y.L.); 2 School of Physics and Astronomy, Cardiff University, Cardiff CF24 3AA, UK; gongy10@cardiff.ac.uk; 3 Wireless and Optoelectronics Research and Innovation Centre, Faculty of Computing, Engineering and Science, University of South Wales, Cardiff CF37 1DL, UK; kang.li@southwales.ac.uk; 4 Harbin Institute of Technology, Harbin 150001, China; weiyuchen@hit.edu.cn (Y.W.); 21S005003@stu.hit.edu.cn (Z.L.); gao_zhiqiang@hit.edu.cn (Z.G.); 5 School of Physical Science and Technology, Suzhou University of Science and Technology, Suzhou 215009, China; wjk31@163.com (J.W.); guozhen.liu@hotmail.com (G.L.); jlyao@usts.edu.cn (J.Y.); jugao@hku.hk (J.G.); 6 School for Optoelectronic Engineering, Zaozhuang University, Zaozhuang 277160, China

**Keywords:** thermal emitter, electro-optical conversion, metamaterials, measurement system

## Abstract

In this study, a multifunctional high-vacuum system was established to measure the electro-optical conversion efficiency of metamaterial-based thermal emitters with built-in heaters. The system is composed of an environmental control module, an electro-optical conversion measurement module, and a system control module. The system can provide air, argon, high vacuum, and other conventional testing environments, combined with humidity control. The test chamber and sample holder are carefully designed to minimize heat transfer through thermal conduction and convection. The optical power measurements are realized using the combination of a water-cooled KBr flange, an integrating sphere, and thermopile detectors. This structure is very stable and can detect light emission at the μW level. The system can synchronously detect the heating voltage, heating current, optical power, sample temperatures (both top and bottom), ambient pressure, humidity, and other environmental parameters. The comprehensive parameter detection capability enables the system to monitor subtle sample changes and perform failure mechanism analysis with the aid of offline material analysis using scanning electron microscopy, energy dispersive X-ray spectroscopy, and X-ray diffraction. Furthermore, the system can be used for fatigue and high-low temperature impact tests.

## 1. Introduction

**Figure 1:**
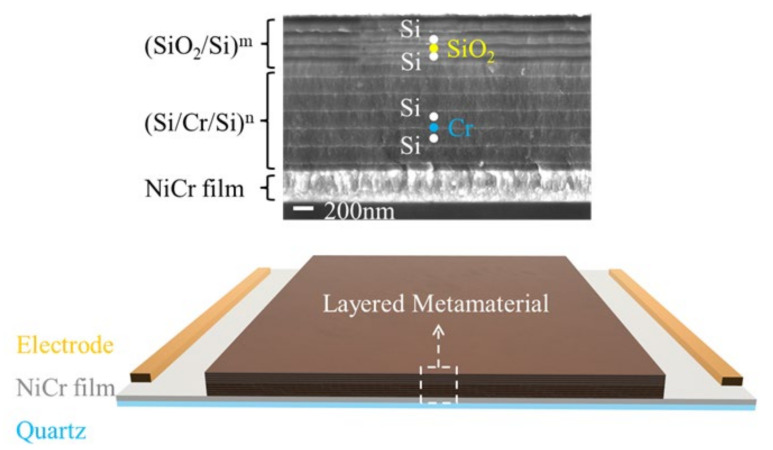
A schematic of the LMM thermal emitter with integrated electric heating film.

Infrared, thermal-emitter-based, layered metamaterials (LMMs) have many advantages over traditional emitters [1,2,3], such as adjustable wavelengths, low visible-light emission, and high efficiency. This type of emitter has broad applications in infrared monitoring [4], gas sensing [5], and radiation cooling [6], and is the research hot-spot in the energy [7,8,9] and sensing fields [10,11,12]. Light emission-correlated tests for LMM are usually performed on bulky external heaters [1,13,14]. However, considering the demand in the sensing field, the LMM thermal emitter is best equipped for these tests, with an integrated heater [15,16,17,18], such as the microhotplates or integrated electric heating films used in the microelectromechanical systems. Recently, we designed an integrated LMM infrared thermal emitter [19], as shown in Figure 1. A quartz substrate is applied at the bottom of the sample coated with a NiCr alloy film. The film is used as a heater and as a light reflector. Two one-dimensional photonic lattices (Si/Cr/Si)^m^ and (SiO_2_/Si)^n^ are located at the top, and these are combined to achieve selective emissions in the middle and far infrared ranges. Here, m and n represent the number of the periods of the two lattices. However, there is no existing test system that can accurately calibrate the electro-optical conversion (EOC) efficiency of an integrated LMM thermal emitter. In addition, whether used for radiation heating or sensing, the emitter will face a very complex environment. However, the existing test system has no atmospheric control capability and can only perform tests under vacuum [9,13] and in atmospheric conditions [14,20]. A test system that can accurately control the gas composition, pressure, and humidity is necessary for environmental simulations to study the influence of environmental parameters on the performances of the infrared thermal emitter. Finally, the research conducted thus far on the LMM emitter is still in the early stage of its development, and many technical issues, particularly the stability of the structure, must be improved [13,21]. This requires the testing system to be able to conduct various types of fatigue tests and sample failure mechanism analyses.

Based on the above requirements, we designed a multifunctional, LMM thermal emitter performance test system based on vacuum technology. The core function of the system is to test the EOC efficiency of the integrated LMM emitter. We used a high-precision programmable power supply to ensure the control accuracy of the input of electrical power. Referring to the light emission characteristics, we designed a combination of a water-cooled KBr flange, an integrating sphere, and a high-precision thermopile detector to ensure the optical power testing accuracy. Additionally, we reduced the thermal conduction and convection of the sample to ensure the testing accuracy of the EOC efficiency measurements. Another advantage of this system is that it is able to synchronously detect multiple sample and environmental parameters. Using this feature in conjunction with offline materials characterization methods, such as X-ray diffraction (XRD), scanning electron microscopy (SEM), and energy dispersive spectroscopy (EDS), the failure mechanism of the samples can be determined. Finally, high and low temperature impact tests were performed on samples in different environments to study their stabilities. This high-precision, multifunctional, atmospheric-controlled EOC efficiency test system can be applied to the research evaluations of the integrated LMM thermal emitter and can be used in diverse applications in other fields requiring photoelectric or EOC measurements that use items such as light-emitting diodes, photoelectric sensors, and infrared gas sensing.

## 2. System Design

**Figure 2:**
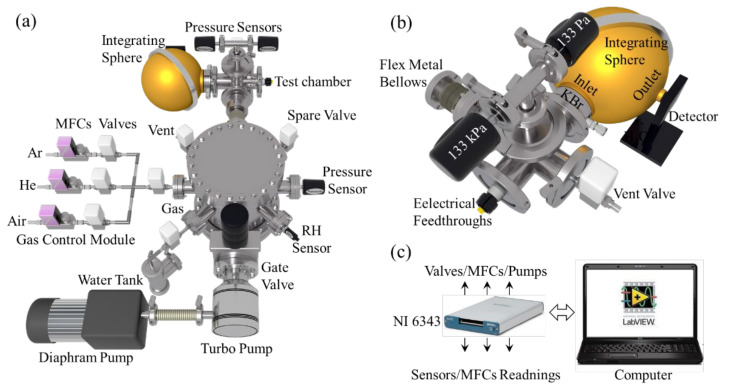
A schematic of the multifunctional electro-optical conversion efficiency measurement system. (**a**) An overview of the environmental control and EOC test modules, (**b**) the back view of the EOC test module, and (**c**) the system control method.

The system consists of three modules: the environmental control, the EOC test, and the system control. Figure 2a shows the top view of the environmental control and EOC test modules, Figure 2b shows the rear view of the EOC test module, and Figure 2c presents the schematic of the system’s control method. We first introduced the environmental control module. The main body of the module is a stainless steel CF200 vacuum chamber, with eight CF flanges welded in the middle. The functions of each flange are marked. A high-vacuum gate valve is installed on Flange 1 (bottom flange, as shown in the figure), which is equipped with both a turbomolecular pump and a roughing pump to provide the system with a high-vacuum environment. It is noteworthy that the selected turbomolecular pump uses damping bearing technology, which is resistant to gas impact and is associated with minimal vibration responses.

Flanges 2 and 3 are equipped with a relative humidity sensor (Honeywell HIH-4000) and a full-range vacuum gauge (Agilent FRG700), respectively. The relative humidity sensor is used when humidity control is required. Flange 5 is used to connect the EOC test module, whose structure will be described in Section 3. The other four flanges are fitted with SMC XSA-series high-vacuum solenoid valves. Among them, Flange 4 is the spare valve used for connections to external systems. Flange 6 is the system ventilation valve. Flange 7 is connected to the gas control module, which is composed of high vacuum solenoid valves and mass flow controllers for controlling the flow of argon, synthetic air, and helium gases. Flange 8 is connected to a water chamber, which is a KF40 three-way cross fitting filled with deionized water. When the valve is open, water vapor can be introduced slowly into the system to humidify the gas mixture. The water chamber and CF200 main chamber are equipped with a heating belt and a type-K thermocouple (TC) for temperature control.

Figure 2b presents the rear view of the EOC test module whose main body is a KF50 5-way cross fitting. The left flange is fitted with a flexible welded bellow for shock absorption and is connected to the environmental control module. The right flange is a high-vacuum solenoid valve for chamber ventilation. The upper flange is mounted with two absolute pressure sensors (Setra 730, and 761) with the rated value ranges of 0–133 Pa and 0–1.33 × 10^5^ Pa, respectively. The front flange is equipped with a hermetically sealed electrical connector for accessing electrical power and for TC signal transmission. The rear flange is connected with a water-cooled KBr vacuum flange, followed by an integrating sphere and an optical power detector.

To realize the ambient composition and humidity control, one should close the gate valve and pumps after the high-vacuum condition is attained. Test gas is then fed into the system through the atmospheric control module. The gas pressure is monitored by a combination of absolute pressure sensors mounted on the test chamber. This type of vacuum gauge has a reading accuracy of ±0.25% and a resolution that is greater than 1 × 10^−4^ of the full scale. Thus, the combination of two pressure sensors can cover the range from 1.3 × 10^−2^ Pa to 1.3 × 10^5^ Pa, fully meeting the test requirements. Most importantly, the readings of these two capacitive pressure sensors are not affected by the type of gas used and the sensors are compatible with a wide range of gases, in addition to water vapor. Although the full-range vacuum gauge mounted on Flange 3 has a wider detection range (5 × 10^−7^–1 × 10^5^ Pa), its reading is sensitive to the type of gas detected. Therefore, this gauge is turned off when the atmospheric composition and the humidity needs to be controlled.

A LabVIEW program controls the system. During testing, the environmental parameters, such as the pressure and humidity of each chamber, the voltage, current, surface, and bottom temperatures, and the electrical and optical powers of the sample, are transmitted to the computer and recorded in real time. System actuators, such as mass flow controllers, valves, and pumps, are controlled by the LabVIEW program according to different setpoints. The comprehensive parameter monitoring capability and automatic testing process provide the system with various testing functions and strong sample compatibility. The system functions are described in detail in the following sections.

## 3. EOC Efficiency Measurements

**Figure 3:**
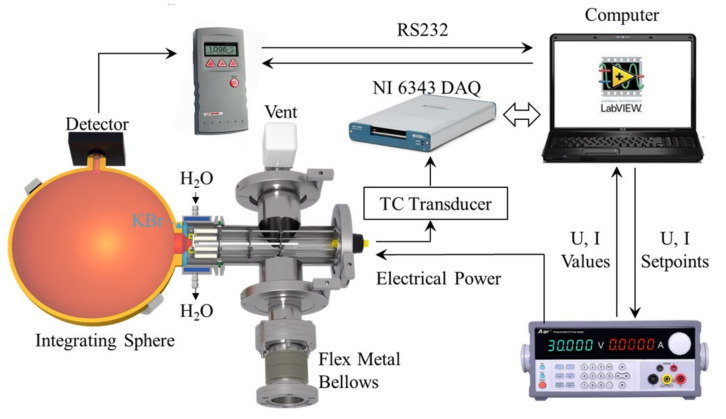
A schematic of the electro-optical conversion (EOC) efficiency measurement mechanism.

**Figure 4:**
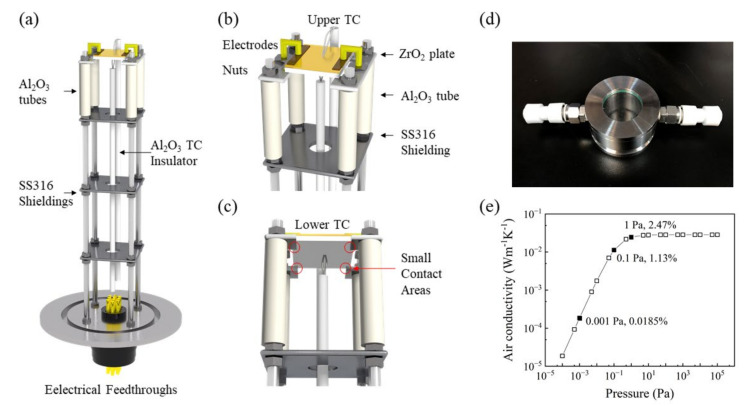
(**a**–**c**) A schematic of the sample holder used for EOC measurements, (**d**) a photograph of the water-cooled KBr flange, and (**e**) the relationship between the chamber pressure and the air conductivity.

The main function of the system is the performance of EOC efficiency measurements. The principle of measurement is explained schematically in Figure 3, in which both the test chamber and the integrating sphere are shown in cross-sectional views to allow the display of the optical path. As shown in the figure, a sample holder is installed throughout the entire test chamber. Its detailed structure is shown in Figure 4a–c. The sample under test is installed at the left end of the sample holder, close to the water-cooled KBr vacuum flange. Because the light emitted by the integrated LMM sample has a certain divergence, we placed an integrating sphere next to the atmospheric side of the KBr flange. The light introduced into the sphere is reflected many times by the highly reflecting diffuse surface of the inner wall. A high-precision thermopile sensor (Ophir 3A-SH), which was installed at the exit of the integrating sphere, detected a small fraction of this light beam. To calculate the real optical path of the sample, the reading of the power meter is divided by the transmission efficiency of the integrating sphere.

In a typical test process, the system first sends the heating voltage and current to the programmable direct current (DC) power supply through the LabVIEW program. Meanwhile, the true values of the voltage and current are transmitted back to the computer to obtain the electrical power. The heating power passes through the electrical feedthroughs into the test chamber to heat the sample and make it glow. The diverging light passes through the KBr flange into the integrating sphere and becomes uniformly distributed after multiple reflections. The optical power is then detected by the thermopile sensor, read by the power meter (Ophir Nova), and is finally sent back to the computer. The temperature signal of the sample is transmitted via the electrical feedthroughs and converted into an analog signal using a temperature transducer. Thereafter, the temperature signal is measured by the NI USB-6343 data acquisition (DAQ) card and recorded as the temperature value of the sample.

**Figure 5:**
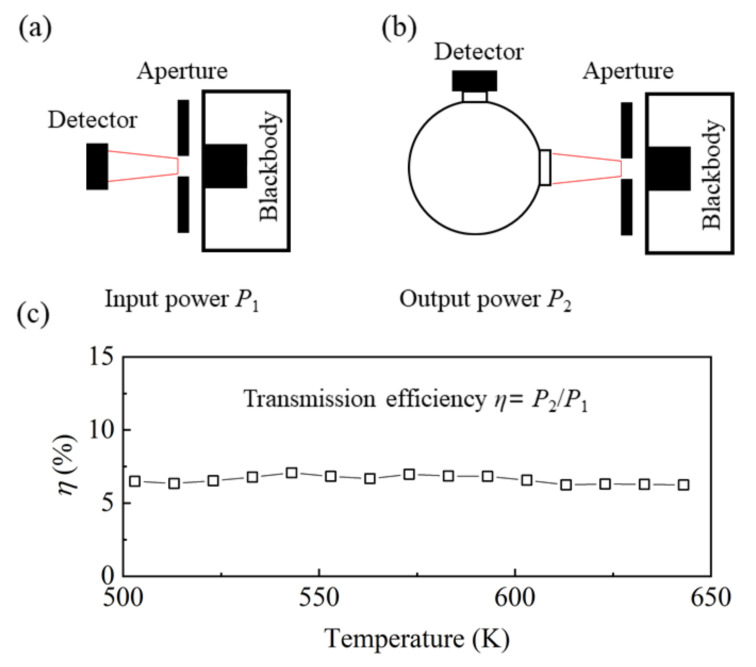
(**a**,**b**) A schematic of the integrating sphere transmission efficiency calibration method, (**c**) the transmission efficiency of the integrating sphere as a function of the blackbody temperature.

To achieve accurate EOC testing, the following three aspects must be considered: the electrical power control accuracy, the optical power detection accuracy, and the reduction of sample heat loss. We used a high-precision programmable DC power supply to control the electrical power fed to the sample. Its current resolution was 0.1 mA and the voltage resolution was 1 mV, which is adequate for EOC testing. For optical power testing, we used a combination of the water-cooled KBr vacuum flange, the integrating sphere, and high-precision thermopile sensors. The transmission spectral range of the KBr window was in the range of 0.25–20 μm, thus meeting the testing requirements of most infrared emitting devices. Furthermore, we used a high-power chiller to cool the KBr flange. As shown in Figure 3, the sample was completely wrapped in the water-cooled flange, effectively reducing the impact of the hot samples on the sealing materials. Among three commonly used light-intensity sensors, i.e., photodiode, thermopile, and pyroelectric sensors, the thermopile sensor offers the best linearity and stability and is insensitive to ambient temperature variation. On the other hand, the signal of the photodiode sensor is prone to saturate at a very low light intensity, whereas the pyroelectric sensor is very expensive and particularly suitable for high-frequency optical signal measurements. The Ophir 3A-SH thermopile sensor used in this study has a detection wavelength in the range of 0.19–20 μm, a power range of 10 μW–3 W, and a noise of 1 μW, which is adequate for measuring the weak light signal that was dispersed by the integrating sphere. The transmission efficiency of the integrating sphere must be calibrated before the test. Figure 5 shows a schematic of the calibration process. The light emitted by the sample was simulated using a commercial blackbody and an aperture. The hole size of the aperture is the same as that of the sample. The first step is to test the optical power at the entrance of the integrating sphere. As shown in Figure 5a, the power meter is placed in front of the aperture, which is at the same position as the entrance of the integrating sphere. The measured light intensity *P*_1_ is recorded as the power at the entrance of the integrating sphere. The integrating sphere was then added to the optical path, and the detector was placed at the exit of the integrating sphere to obtain the light intensity *P*_2_, Figure 5b. The integrating sphere transmission efficiency *η* is equal to the quotient of *P*_2_ and *P*_1_, i.e., *η* = *P*_2_/*P*_1_. The infrared radiation of the samples at different temperatures can be simulated by changing the temperature of the blackbody. As shown in Figure 5c, the integrating sphere transmission efficiency is ~0.065% and is rather stable at different temperatures. In subsequent tests, the light intensity measured by the power meter was divided by the transmission efficiency of the integrating sphere to convert it to the true optical power of the sample.

The final step is to minimize the heat loss of the sample due to thermal conduction and convection. Thermal conduction is mainly caused by components that are directly in contact with the tested sample, including the thermocouples (TC), electrodes, and ceramic plates on which the sample was mounted, as shown in Figure 4a–c. For the TC, we used the Omega CHAL-010 k-type bare TC, with a diameter of only 0.25 mm. This type of unsheathed TC was more sensitive to temperature changes. We also attempted to use thinner (0.05 mm) and thicker (0.8 mm) TCs. In the former case, it was difficult to establish a close contact with the sample, while the latter would lead to increased heat dissipation from the sample; hence, the measured temperature was much lower than the actual value. The electrodes were made of gold-plated copper with a diameter of 2 mm and a rated power of 10 A. We choose ZrO_2_ plates to support the tested samples. As shown in Table 1, the heat capacity and thermal conductivity of the ZrO_2_ plates were only 58.3% and 6.6% of the respective values for Al_2_O_3_ [22]. Moreover, the ZrO_2_ plates have a high-melting point and are very stable in adverse environments. Furthermore, the ZrO_2_ plate was cut by laser, with only four points of contact with the sample, as shown in Figure 4c. With the use of the aforementioned methods, the heat loss via heat conduction was reduced considerably.

The bottom portions of the ZrO_2_ plates consist of four ceramic tubes for electrical and thermal insulation, fixed by four stainless steel screws. In addition, three polished 316 stainless steel discs were mounted on the screws. The main function of these discs is to reduce the influence of sample thermal radiation on the internal components of the chamber.

(1) $$Q_{n}=\frac{1}{n+1}\left[5.67\times 10^{-8}A\frac{\varepsilon }{2-\varepsilon }\left(T_{1}^{4}-T_{2}^{4}\right)\right]$$

According to Equation (1), the radiative heat transfer *Q_n_* between the cold and hot plates in the vacuum chamber is mainly determined by the temperatures of the two plates (*T*_1_ and *T*_2_), the average emissivity *ε* of the plates, and the number of radiative screens implemented between the two plates, *n* [22]. When *n* = 3, the radiative heat transfer from the hot end to the cold end reduces to 1/4 of its initial value. These stainless-steel radiative screens protect the fragile parts of the test chamber, specifically the electrical feedthroughs and rubber O-rings.

The final operation involves the reduction of the thermal convection caused by the test ambient. For a closed chamber comprising two plates, the relationship between the thermal conductivity of the air, temperature, and pressure is expressed in Equation (2)

(2) $$\kappa_{air}=\kappa_{0}\times \frac{1}{1+\frac{7.6\times 10^{-5}}{p\times \frac{d}{T}}}$$

where $\kappa_{air}$ is the thermal conductivity of the residual air, $\kappa_{0}$ is the thermal conductivity of the air at room temperature and atmospheric pressure, *d* is the spacing between plates, and *T* is the average temperature of the two substrates [23]. When the cavity temperature is 300 K, cavity length is 13 cm, and air thermal conductivity is 0.0284 Wm^−1^ K^−1^ at atmospheric pressure, according to Equation (2), the calculated relationship between the thermal conductivity of residual air and its pressure is shown in Figure 4e. The thermal conductivities at 1, 0.1, and 0.001 Pa are marked in the figure, which correspond to the ultimate pressure of the dry scroll pump, rotary vane pump, and turbomolecular pump, respectively. As shown, when the air pressure is 0.001 Pa, its thermal conductivity is only 0.0185% of the atmospheric pressure. The abovementioned heat conduction and exchange control scheme can effectively reduce the heat loss of the sample and improve the accuracy of the EOC efficiency test of the sample.

**Figure 6:**
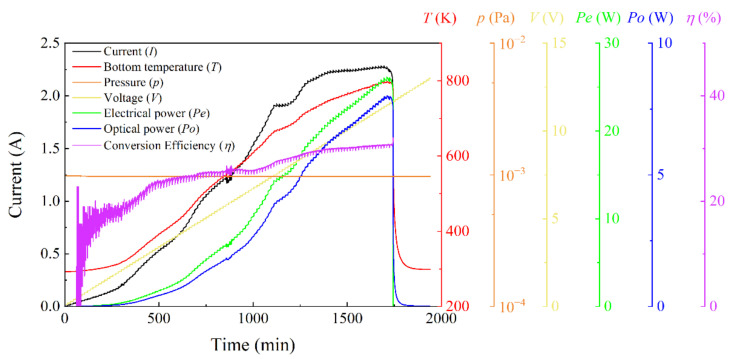
The temporal evolutions of the input voltage (*V*), current (*I*), bottom temperature (*T*), chamber pressure (*p*), electrical power (*P*_e_), radiated optical power (*P*_o_), and EOC efficiency (*η*) of an LMM thermal emitter.

Figure 6 presents the result of the EOC efficiency test of an LMM thermal emitter measured by this system. The sample is composed of a 3 mm quartz substrate, 300 nm NiCr alloy heating film, and a composite LMM of (Si/Cr/Si)^m^/(SiO_2_/Si)^n^ used as an infrared emitting layer. During the test, the heating voltage was increased by 0.1 V every 15 min to ensure that the sample was warmed at a slow rate to allow an adequate heat exchange with the surrounding environment. The heating current, temperature, test chamber pressure, electrical power, and optical power of the sample were recorded in real time. As shown in the figure, with the increase in the temperature, the EOC efficiency of the sample increased gradually and finally stabilized at ~30%. However, the temperature of the sample was still low, and there is still room for improvement. With the additional increase in temperature, the proportion of heat loss will likely be further reduced, and the infrared emission power of the samples, as well as the EOC efficiency, is expected to be improved. Therefore, it is particularly important to study the failure mechanism of the sample to increase its temperature limit.

## 4. Failure Mechanism and Fatigue Test

The system can measure the electrical and optical parameters, as well as the atmospheric pressure, synchronously, which provides a fundamental basis for the analysis of the failure mechanism of the samples. In the following section, we discuss ways to analyze the failure mechanism of the samples based on two case studies, one involving an integrated LMM thermal emitter and the other a separate electrical heating film.

**Figure 7:**
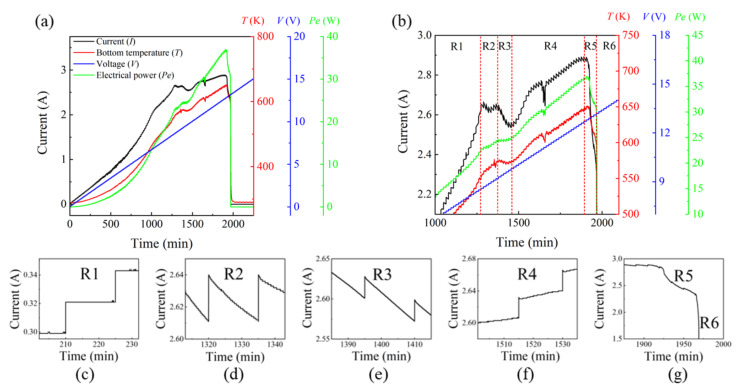
(**a**) The failure test of a typical integrated LMM thermal emitter. (**b**) An enlarged view of the failure process, which is divided into six regions denoted by R1–R6. (**c**–**g**) The enlarged views of the current evolution in each region.

The first case involved the failure mechanism of the LMM thermal emitter during its operation in air. Because the outmost layer of the LMM is silicon dioxide, which is used to protect the sample from being oxidized, the LMM emitter is designed to work at different ambient conditions. We tested the failure process of dozens of samples, and the most typical process is shown in Figure 7; this includes all typical evolution processes. During the test, the heating voltage increased by 0.1 V every 15 min. In Figure 7a, the variations of current, temperature, and electrical power are plotted as functions of the heating time. Figure 7b provides an enlarged view of the failure process. Among all of these parameters, the heating current of an LMM emitter is most relevant to the failure mechanism. According to the changing details of the current, the entire test process can be divided into six regions, marked as R1–R6 in Figure 7b. Figure 7c–g shows the details of the current changes in each region. Before we can execute the analysis, we need to define the sample structure (Figure 1). At the bottom of the sample is a 3 mm quartz substrate on which there is a 300 nm NiCr electrical heating film and an LMM composed of (Si/Cr/Si)^m^/(SiO_2_/Si)^n^. As shown, only the NiCr film is conductive, whereas the quartz substrate and LMM are nonconductive at room temperature.

In Region 1, the sample current equals the current in the NiCr film, i.e., I_sample_ = I_NiCr_, which increases monotonically as a function of the voltage. Figure 7c shows that when the voltage increases, the I_NiCr_ increases concomitantly and stabilizes rapidly. The electrical power and temperature of the sample also increases in a step-by-step manner.

In Region 2, the sample current I_NiCr_ reaches saturation and does not increase when the voltage increases. Figure 7d shows that the current slowly decreases to its saturated value after a sudden increase. Although the current does not change, the voltage gradually increases; thus, the electrical power and the sample temperature also slowly increase in Region 2.

In Region 3, the sample current I_NiCr_ decreases as the heating voltage increases. Accordingly, the current drops rapidly after a current surge, Figure 7e. This is because the electrical power of the NiCr film is in a saturated state, and a sudden increase in the voltage to 0.1 V leads to the increase in the sample’s temperature. The intensified lattice vibration of the NiCr film at elevated temperatures increases the film’s resistivity owing to electron scattering, and the saturation current gradually decreases. The sample electrical power and temperature remain unchanged in this region.

In Region 4, the sample current increases rapidly as the voltage increases. The current continues to rise slowly after the current surge, Figure 7f. Because quartz does not conduct electricity, the current flowing through the NiCr film reaches saturation and starts to decline; we believe that this is due to the emergence of a new conductive channel in the layered MM (Si/Cr/Si)^m^/(SiO_2_/Si)^n^. Thus, the total current of the sample I_sample_ = I_NiCr_ + I_LMM_. The structure of the LMM is rather complex, and the lower (Si/Cr/Si)^n^ photonic crystal is more prone to become conductive at elevated temperatures due to the diffusion of Ni, Cr into the Si layer [24,25,26]. The electrical power and temperature of the sample also increases rapidly within this range.

After the LMM becomes conductive, the sample finally reaches the critical point and eventually breaks down. The breakdown process is further divided into Regions 5 and 6, which are more noticeable in Figure 7g. Moreover, the lowest current of Region 5 is slightly lower than that of Region 3. This indicates that the LMM of the sample is damaged at first, and the I_LMM_ rapidly decreases to zero, that is, I_sample_ = I_NiCr_ again. Because the temperature of the NiCr film in Region 5 is higher than that of Region 3, the resistivity is higher and the saturation current is lower. After losing the protection of the LMM, the NiCr electrical heating film oxidizes rapidly and the sample current quickly decreases to zero in Region 6. If the sample is tested under vacuum conditions, the optical power of the sample and the pressure in the chamber shows a sudden increase in Region 5. This indicates that the damage to the sample is accompanied by flickering, and part of the LMM is vaporized. The addition of quadrupole mass spectrometers can assist in the study of the sample failure mechanisms.

The failure mechanism analysis described above provides the following guidelines for sample improvement: (1) The saturation current of the NiCr film is relatively small, and thus results in a low-sample temperature and a low-EOC efficiency. It is helpful to increase the thickness of the NiCr film, or to replace it with another electrical heating film that is more resistant to high temperature and oxidation. (2) Buffer layers such as Ta_2_O_5_ and HfO_2_ should be inserted between the NiCr heating film and the LMM to prevent interdiffusion between the layers and to improve the operating temperature threshold of the LMM. (3) Reducing the thickness of the quartz substrate and fabricating the electrical heating film on the other side of the LMM may be helpful in eliminating the interlayer diffusion between the heating film and the LMM. (4) Working with gases exhibiting higher thermal conductivities may improve the surface heat dissipation of the LMM, likely improving the lifetime.

**Figure 8:**
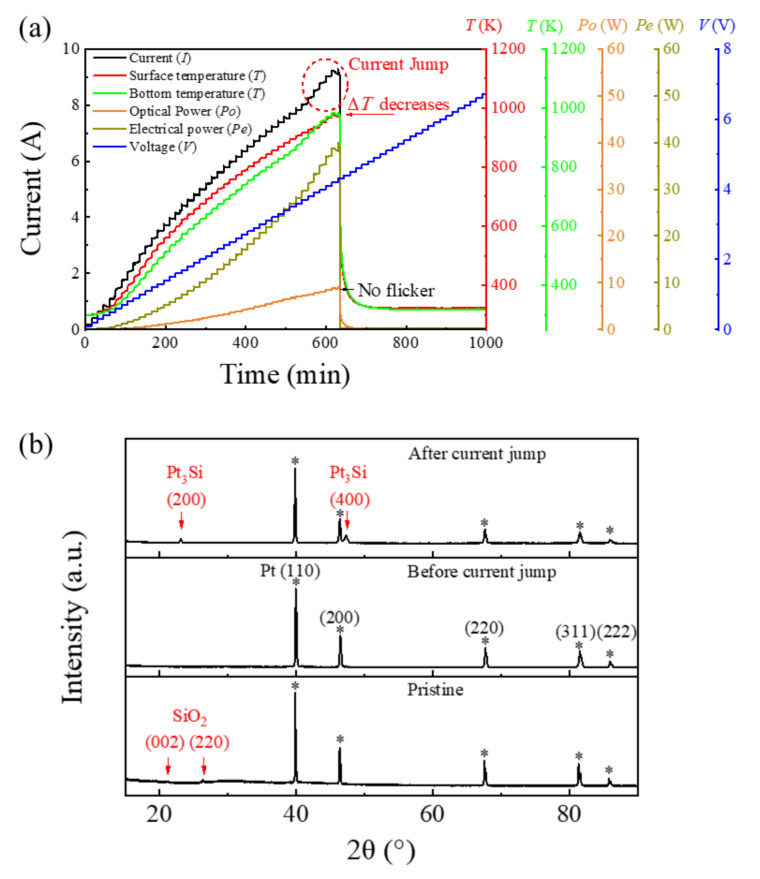
(**a**) The failure test of the platinum (Pt) electric heating film used in an LMM emitter. (**b**) The X-ray diffraction patterns of the Pt films in a pristine state, as well as before and after the current jump. (The * stands for diffraction peaks originated from Pt metal.)

Based on the above arguments, a thick Pt film was prepared as a new type of electrical heating film by using screen printing. The thickness of the quartz substrate was also reduced to 0.2 mm in the tests described below. Figure 8a shows the curves of the heating current, the surface and bottom temperatures, electrical power, and optical power of Pt thick film as a function of the heating time. The voltage increased by 0.1 V every 15 min during the test. Owing to the simple structure, the current evolution of the Pt thick film was also much simpler than the integrated LMM emitter used in the study of Case 1. The current increases steadily with the voltage in most of the evolution processes. In the last few cycles, the sample current yielded an abrupt increase, and the sample then broke down. Simultaneously, the current jump of the sample was accompanied by the relative change in temperatures on the surface and bottom of the sample. Before the current jump, the surface temperature of the sample was consistently ~30 K higher than the temperature at the bottom. However, in the process of current jump, the temperature on the back side increased rapidly and finally became consistent with the temperature on the film surface. Therefore, this process must be accompanied by the improvement of thermal contact between the thick Pt film and the quartz substrate.

**Figure 9:**
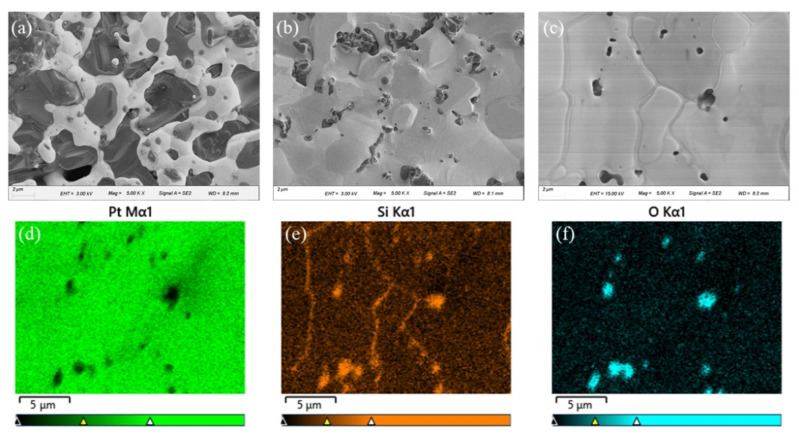
Scanning electron microscopy images of the Pt electric heating films. (**a**) The pristine film, (**b**) before the current jump, and (**c**) after the current jump. (**d**–**f**) Energy dispersion X-ray spectroscopy mapping of the Pt, silicon, and oxygen elements of the Pt electric heating film after the current jump.

Because it is difficult to determine the cause of failure based on the above electrical and thermal data, we conducted XRD, SEM, and EDS characterizations of the intrinsic Pt film and the Pt film before and after the current jump; the results are shown in Figure 8b and Figure 9. The pristine Pt film is made of a slurry composed of Pt particles and glass powder. In the SEM image, glass particles (sizes of the order of μm) were embedded between finer Pt particles. The Pt forms a sinuous conductive channel, as shown in Figure 9a. At this point, the interface between the two phases is clear. In the XRD pattern, there are weak quartz peaks (Powder Diffraction File (PDF) 45-0130) and wide amorphous peaks in the range of 20°–30°, which means that the glass powder is partially crystallized. Concurrently, all XRD diffraction patterns have five very strong Pt diffraction peaks (PDF 04-0802), indicating that the Pt is highly crystallized.

For the samples before the current jump, the quartz peaks in the XRD patterns disappeared. The SEM images showed that the surface coverage of the Pt increased considerably, and only a small part of glass grains were exposed. This result is consistent with the details of the current evolution curve depicted in Figure 8a. The detailed current evolution in Figure 8a shows that the slope, i.e., the film resistance, is changing continuously. When the current is less than 4 A, the slope of the current curve increases gradually, that is, the sample resistance decreases gradually. When the current is higher than 4 A, the slope of the current curve decreases gradually, that is, the sample resistance increases gradually. Pt is a well-known temperature-sensitive material with a linear temperature coefficient of resistance. Typically, as the temperature increases, the resistance should increase linearly, that is, the slope of the current curve should remain constant. However, the electrical conductivity of the Pt film is enhanced by the increasing Pt surface coverage, reducing the resistance of the film. Therefore, the change in the resistance of the Pt thick film was significantly different from that of the typical Pt temperature sensor owing to the synergistic effects of the increase in temperature and surface coverage.

For the Pt film after the current jump, the XRD pattern shows the diffraction peaks that can be assigned to Pt_3_Si (PDF 17-0670), implying that Pt may react with the quartz substrate or glass powder to generate Pt silicide [27]. This hypothesis was confirmed by the SEM and EDS results. Figure 9c shows that after the current jump, the coverage of the Pt film further increased, with only a few holes remaining. However, the Pt thick films yielded unusual, significant grain boundaries. Figure 9d–f shows the distribution of the platinum, silicon, and oxygen elements within the range of Figure 9c. As shown in Figure 9d, the surface coverage of the Pt is extremely high, and Pt is absent only at the hole position. The distribution of oxygen is completely complementary to the distribution of Pt, i.e., oxygen only exists in the hole position. This oxygen signal comes from the silicon oxide in the glass powder, as shown in Figure 9f. In addition to in the pores, silicon is also found at the grain boundaries (Figure 9e), whereas no peaks of oxygen were found at these boundaries. Therefore, the final increase in the current is caused by the formation of Pt silicide. This finding could be attributed to the considerably lower resistivity of Pt silicide than that of silicon oxide combined with the greatly enhanced electrical current [28]. Considering that the quartz substrate was also made of SiO_2_, it may react with Pt to generate silicide at the interface between the Pt film and the substrate. Consequently, the thermal contact between the Pt film and the substrate was enhanced considerably [28], and the difference between the film surface temperature and the temperature at the bottom decreased rapidly, as shown in Figure 8a. This process shows that the multiparameter synchronous test, combined with the offline materials characterization, can help to determine the failure mechanism of the samples and can provide a reference for changing sample performance.

**Figure 10:**
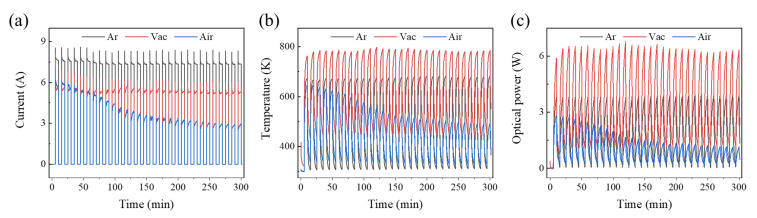
The environmentally controlled high- and low-temperature test outcomes of the silicon-based heating plate used in an integrated LMM emitter in different gas environments. (**a**–**c**) The dynamic responses of the current, temperature, and optical power.

Finally, we conducted high- and low-temperature impact tests with the sample in argon, vacuum, and in humid air (relative humidity was equal to 35%). The atmospheric control method was described in Section 2. The sample used is a monocrystalline silicon heating plate. The heating voltage of the sample switched between 5 V and 0 V every 5 min. The currents, temperatures, and optical powers of different test rounds are shown in Figure 10a–c. A comparison of the three atmospheres showed that the upper and lower limits of current, temperature, and light power were stable in argon and under vacuum. However, when tested in humid air, the sample current decreased significantly in the first 10 cycles, gradually stabilizing. The sample temperature and optical power showed the same attenuation trend. This result indicates that the sample electrodes were prone to oxidization at high temperatures. The test under vacuum and argon also led to large discrepancies. The electrical power of the sample under vacuum was lower than that in argon, but the upper and lower limits of the sample temperature and the optical power were much higher than the test results in argon. This indicates that argon gas has a high specific-heat capacity and thermal conductivity, leading to a significant increase in the thermal loss of the sample. It is noteworthy that the sample current showed obvious spikes due to the sample temperature variation. At the beginning of each cycle, the sample current first increased to its maximum due to the sudden rise of heating voltage. Then the sample temperature increased slowly due to the Joule heating effect. As a result, the sample resistance/current increased/decreased accordingly, finally stabilizing at a certain level depending on the conditions of testing environment. The research described above indicates that, as a form of fatigue test, high- and low-temperature impact tests under different ambient conditions are of great importance to the study of the stability of LMM devices.

## 5. Conclusions

A multifunctional testing system was constructed for the characterization of integrated LMM thermal emitters based on vacuum technology. The primary function of the system was the realization of the high-precision measurement of the EOC efficiency of the device. For this purpose, we improved the control accuracy of the electric power of the sample based on the use of a high-precision programmable power supply. We achieved high-accuracy measurements of the sample optical power using of a combination of a water-cooled KBr vacuum flange, an integrating sphere, and a high-precision thermopile detector. Finally, with the careful design of the sample holder and the use of high-vacuum test conditions, the thermal conduction and convection was reduced considerably, which is important for the accurate evaluation of the EOC efficiency. The system can synchronously record various parameters of the sample. The detailed and comprehensive test data provided a fundamental basis for the analysis of the sample failure mechanism. The failure mechanism of the integrated LMM samples was analyzed based on the variation of the sample current, temperature, electrical power, optical power, and ambient pressure. The failure mechanism of the thick Pt film was studied with the aid of offline XRD, SEM, and EDS characterizations. Finally, the sample resistance to high- and low-temperature shocks was tested in different atmospheres. The results were strongly related to the test environments, proving the importance of testing for device fatigue. The system was composed of standard vacuum components and was automatically controlled by the LabVIEW program. Compared with the existing system, this system showed high test accuracy and was multifunctional, e.g., the control of the atmosphere and the failure mechanism analysis could be realized; the system was also capable of performing sensor fatigue tests in different modes. This system cannot only be used in the research of integrated LMM emitters, but it also has applications in other fields which require photoelectric or electro-optical conversion measurements, as well as infrared gas sensing. In the future, we will modify the system to adapt to reflectivity and transmissivity spectra measurements, as well as optical emissivity and thermal radiation measurements at elevated temperatures, to get a deeper and more complete description of the light emission characteristics of the LMMs. Another improvement would be the integration of a quadrupole mass spectrometry system for online failure mechanism analysis.

## Figures and Tables

**Table 1:** The thermal properties of the typical insulating materials used in under vacuum.

Materials	Melting Point (K)	Heat Capacity (Jkg^−1^ K^−1^)	Thermal Conductivity (Wm^−1^ K^−1^)	
			373 K	773 K
Al_2_O_3_	2323	774	30.3	11.0
CaO	2873	766	15.3	8.1
MgO	3073	938	36.2	14.0
SiO2	1883	737	0.9	1.6
ZrO_2_	2950	452	2	2.1

## Data Availability

Not applicable.
